# Interprofessional Oral Health Education Improves Knowledge, Confidence, and Practice for Pediatric Healthcare Providers

**DOI:** 10.3389/fpubh.2017.00209

**Published:** 2017-08-14

**Authors:** Devon Cooper, JungSoo Kim, Karen Duderstadt, Ray Stewart, Brent Lin, Abbey Alkon

**Affiliations:** ^1^School of Dentistry, UCSF, San Francisco, CA, United States; ^2^UCSF School of Nursing, San Francisco, CA, United States

**Keywords:** oral health, prevention, children, early childhood, interdisciplinary, underserved population/multicultural

## Abstract

Dental caries is the most prevalent chronic childhood disease in the United States. Dental caries affects the health of 60–90% of school-aged children worldwide. The prevalence of untreated early childhood dental caries is 19% for children 2–5 years of age in the U.S. Some factors that contribute to the progression of dental caries include socioeconomic status, access to dental care, and lack of anticipatory guidance. The prevalence of dental caries remains highest for children from specific ethnic or racial groups, especially those living in underserved areas where there may be limited access to a dentist. Although researchers have acknowledged the various links between oral health and overall systemic health, oral health care is not usually a component of pediatric primary health care. To address this public health crisis and oral health disparity in children, new collaborative efforts among health professionals is critical for dental disease prevention and optimal oral health. This evaluation study focused on a 10-week interprofessional practice and education (IPE) course on children’s oral health involving dental, osteopathic medical, and nurse practitioner students at the University of California, San Francisco. This study’s objective was to evaluate changes in knowledge, confidence, attitude, and clinical practice in children’s oral health of the students completed the course. Thirty-one students participated in the IPE and completed demographic questionnaires and four questionnaires before and after the IPE course: (1) course content knowledge, (2) confidence, (3) attitudes, and (4) clinical practice. Results showed a statistically significant improvement in the overall knowledge of children’s oral health topics, confidence in their ability to provide oral health services, and clinical practice. There was no statistically significant difference in attitude, but there was an upward trend toward positivity. To conclude, this IPE evaluation showed that offering an interprofessional course on children’s oral health to graduate students in dentistry, nursing, and osteopathic medicine can improve their knowledge, confidence, and practice toward children’s oral health and expand their professional goals to include caring for underserved, minority children.

## Background

Approximately 60–90% of school age children worldwide have experienced dental caries (1). In the United States (U.S.), dental caries are the most prevalent chronic disease in childhood (2). Currently 23% of children from 2 to 5 years of age have dental caries in their primary teeth and the prevalence of untreated caries is 19% (2–4). Children suffer from dental caries at a rate five times greater than asthma in the U.S. (5). To address this public health crisis and growing oral health disparities in children, collaborative efforts among health professionals is critical for dental disease prevention and optimal oral health.

Oral health is commonly defined as the absence of oral disease. In 2016, the Federal Dental International (FDI) Dental World Federation defined oral health more comprehensively. Oral health is the capability to speak, taste, smile, touch, chew, swallow, and express a variety of emotions through facial expressions with confidence and no pain, discomfort, and disease of the craniofacial complex (6).

One of the major contributing factors of oral health disparity is poverty. The U.S. ranks second to last in child poverty in the developed countries (7). In 2008, 4.6 million children in the United States did not receive necessary dental care due to financial hardship (8). In California (CA), approximately 47% of children are growing up in poor families and/or reside in subsidized housing for low-income families (8). Specifically in San Francisco, CA, 40% of children attending schools serving primarily those from low-income households experienced untreated dental caries by the time they entered kindergarten, which is eight times higher than the rate of untreated dental caries in high-income schools (9).

Racial disparities in oral health are also prevalent. In California, Hispanic/Latino (49%), African-American (45%), and Asian (54%) preschool children experience untreated dental decay at rates higher than those of whites (34%) (10). Some racial disparities in oral health can be attributed to cultural differences in feeding and levels of oral health knowledge.

Children are one of the vulnerable and underserved populations that have persistent, systemic obstacles to accessing preventive oral health care (11). As of December 2016, over 35 million children were enrolled in Medicaid (12), a combined state and federal health program to cover medical expenses for individuals with limited income and resources in the U.S. (13). In fiscal year 2014–2015, nearly 5.4 million children aged 0–20 years were enrolled in Denti-Cal, California’s Medicaid dental plan, with numbers showing an increasing trend every year (14).

Over five million children in California are eligible to receive dental care through Denti-Cal; however, the majority of them do not utilize the service. According to the Department of Health and Human Services, from October 2014 through September 2015, only 51.8% of children and teenagers with Denti-Cal attended at least one dental visit (15), suggesting that nearly 50% had not seen a dentist. Children without insurance (30.2%) or with public insurance (27.8%) have higher rates of oral health problems compared to children with private insurance (16.8%) (National Survey of Children’s Health 2011–2012) (16). Uninsured children were the most likely to have never been to a dentist in their life (18.7%) and those with Medi-Cal were the most likely to have never been to a dentist (15.7%) compared to those with other types of insurances (17).

One of the reasons behind the low utilization rates of Denti-Cal is the limited access to local dentists who accept patients covered by Denti-Cal insurance. In a 2016 report by the Little Hoover Commission, at least 5 out of 58 counties in California had no dentists accepting Denti-Cal and numerous other counties had no dentists who are accepting new Denti-Cal patients (15). California has more dental health professional shortage areas than any other state (18). Some reasons for this low number of dentists willing to provide services to patients with Medicaid are because of the program’s low reimbursement rates, frequently missed appointments by patients, and reluctance to treat patients with potentially complex dental issues (9).

Children living in rural areas face greater obstacles in receiving oral health care as many dentists do not live in rural areas where many underserved populations reside (19). Dentists are not evenly distributed geographically (20) and, furthermore, many dentists are not comfortable treating young children under 6 years of age. A 2006 study concluded that a large percentage of dentists did not feel prepared to treat children, especially if the children were very young (21). Professional health sciences schools should be responsible for training graduates to meet the oral health needs of all children. Novel educational programs need to be established urgently to nurture health professionals with the skills and confidence to treat children and to help address the oral health disparity for children.

Unfortunately, recruiting more dental health providers to accept Medicaid and to serve in health professional shortage areas requires policy changes. Recruiting dental health providers will continue to be a challenge in California until changes are made in the Medicaid reimbursement system. According to the Little Hoover Commission, the majority of California’s 31,640 professionally licensed dentists, and a sizeable share of those preparing to become dentists, do not intend to participate in Medicaid (15). Currently, only 29% of California dentists participate in the Medicaid program and 42% of dentists participate nationally. These recruitment challenges demonstrate that dentists cannot provide oral health prevention for all children (15).

## Background and Rationale

Although researchers have acknowledged the various links between oral health and overall systemic health, oral health care usually remains independent from pediatric primary health care (22). In 2011, the Human Resources and Services Administration (HRSA) suggested that primary care practitioners include oral health care services in their primary care practice to help reduce the disparity in preventive dental care for children younger than 5 years old (23). It was suggested that family physicians and pediatric primary care providers should play an increasingly significant role in assessing oral health of children (23). The American Academy of Pediatrics reported that about 90% of infants and children up to 1 year of age have seen a primary care clinician, but less than 2% have been to a dentist (24). These providers may care for a child up to 11 times before the child sees a dentist; thus, “well child” appointments are ideal opportunities to provide oral health assessments, to apply fluoride varnish, and to educate parents on key oral health messages (24). An example of a missed opportunity is the discrepancy between the recommendation that every infant, toddler, and preschooler have a regular application of fluoride varnish to prevent dental caries; yet, it is reported that only 4% of primary care providers are applying fluoride varnish (24). With training and an understanding of the indications and limitations of topical fluoride application, advanced practice nurses, registered nurses, licensed practical nurses, physicians, physician assistants (PAs), and medical assistants, in some states, are allowed to apply fluoride vanish (24).

Healthy People 2020 is the U.S. government’s plan for creating a healthier nation, and some of their objectives are to attain high-quality, longevity free of preventable disease, disability, injury, and premature death; achieve health equity, eliminate disparities, and improve the health of all groups; create social and physical environments that promote good health for all; and promote quality of life, healthy development, and healthy behaviors across all life stages (25). To address these goals, the University of California, San Francisco (UCSF) developed a didactic course with associated clinical experience to train dental care providers and other primary healthcare providers on preventive oral health for young children. Primary healthcare providers can play an essential part and have the chance to counsel their patients on taking their child to the dentist for early disease intervention, especially for young and low-income children (24).

There may be a lack of knowledge about oral health prevention in the care for infants and young children and those with special needs in the training of general dentists, as well as teaching of pediatric providers and other professionals (26). The education and training of dental professionals’ focuses on procedures leaving less time for the interprofessional health and/or social issues. It is not possible to address these knowledge gaps without an integration of dentistry with medicine, nursing, and other health professions. Providing children’s oral health care is an ethical obligation of dental and other health professionals caring for children and working with parents.

There are a limited number of studies on oral health education programs for primary care providers. One study evaluated an oral health education program provided as part of the curriculum in a Masters nursing program for pediatric nurse practitioners (PNP) to increase the number of primary care providers trained on preventive oral health care for young children, particularly those who do not have access to a dental home. A 1-h lecture was given to 30 PNP students by pediatric dental faculty members and a pediatric dental resident based on the First Smiles and American Academy of Pediatrics content (27). The students also participated in a practicum where they practiced the examination techniques and fluoride varnish applications. Pre-tests and post-intervention tests showed positive changes in the students’ knowledge, confidence, and attitudes of oral health skills (28).

Another study consisted of 50 interviews with pediatricians to complete a questionnaire about children’s oral health knowledge. The results concluded that there is a need for more communication between the two specialties of medicine and dentistry to deliver better oral health care to children (29). Another study mailed knowledge questionnaires on children’s oral health to 464 family medicine program directors and 208 completed the questionnaire. The results showed that less than 30% of the program directors felt comfortable with the application of fluoride varnish. The program directors felt this way because of the lack of knowledge on children’s oral health and it was concluded that 95% of family medicine program directors believed oral healthcare knowledge should be a component in the residency training (30).

To help address the oral health needs of young children, an interprofessional oral health course was developed for students in nursing, medicine, dentistry, and osteopathic medicine. The aim of the interprofessional pediatric oral health course was to train health professionals to increase their knowledge of pediatric oral health, increase their clinical competencies in preventive oral health care, educate their pediatric patients and parents on how to maintain good oral health, and to provide primary oral health care to the underserved, vulnerable, and rural communities upon graduation.

## Materials and Methods

An interprofessional practice and education (IPE) oral health elective course for students in dentistry, nursing, and osteopathic medicine was developed by the UCSF interdisciplinary faculty. The course was offered at UCSF for all graduate students for three quarters; each quarter is 10 weeks. During the summer 2016, we piloted tested the IPE course. Over the subsequent three quarters, the course enrolled students at the UCSF School of Dentistry, UCSF School of Nursing, and Touro University College of Osteopathic Medicine students as one of their elective courses. The students signed a consent form, completed demographic questionnaires before the first class, and completed online questionnaires before and after the course. The UCSF Committee on Human Research approved the study’s protocols, consent forms, and evaluation procedures.

The interprofessional elective oral health course included weekly 2-h lectures for 10 weeks. Students were required to attend at least 8 of the 10 sessions to pass the course. The interdisciplinary faculty members are from the UCSF Schools of Dentistry, Nursing, and Medicine. The course covered topics on children’s oral health, barriers on access of care, and addressed disparities in oral health and the needs of low-income communities (see Table 1 of class titles and objectives). Course content was placed on the UCSF learning platform Collaborative Learning Environment website for students to access the syllabus, lecture schedule, lecture slides, articles, resources, and supplementary materials. During the course lectures, students were asked to collaborate in groups of 2–3 on particular oral health issues brought up in the lecture to create an interactive learning environment. Students in the course were from diverse racial and ethnic backgrounds, so collaboration among interprofessional and diverse student groups enriches the class discussion and application to clinical practice. The course also included a clinical component where students observed a pediatric dentist and completed an oral health assessment of a toddler and applied fluoride varnish under the supervision of a dentist. Students were provided with opportunities for community outreach events and clinics they could attend throughout the 10 weeks to complete the clinical requirements. The students were also given a checklist to complete after participating in the two clinical skills sessions, and proof of participation by the supervising dentist was submitted to complete the course requirements.

**Table 1:** Interprofessional course: children’s oral health for primary care providers.

	Topic	Objective
1.	Introduction to Children’s Oral Health and Community Dentistry	To define what dental caries is and what it means to be a community health care provider
2.	The Effect of Cultural and Linguistic Competency and Health Literacy on Access to Oral Health Care	To be made aware of different cultures expectations on health care and that health literacy plays a role in accessing care
3.	Physical Assessment of Oral Cavity and Recognition of Abnormalities	To be able to evaluate and recognize pathology in the oral cavity during regular check-up appointments
4.	Caries Risk Assessment and Disease Prevention	To be able to determine if the child is at high risk for developing dental caries and how to prevent dental caries
5.	Anticipatory Guidance in Pediatric Dentistry	To be able to have an oral health conversation with parents
6.	Infant Oral Health Care, Dental Home, and Referral	To be able to refer infants when their first tooth erupts or when they are 1 year old
7.	The Relationship between Children’s Oral Health and the Overall Systematic Health	To be able to recognize that oral health is connected to overall health
8.	Oral Health in Special Needs and Vulnerable Children	To be able to care for the special needs children and know how to manage them
9.	Management of Orofacial Trauma and Acute Dental Care	To be able to handle a dental trauma and refer when needed
10.	Case Presentations and Final Assessment	To have open class discussion about what has been taught and answer questions about cases that summarize the course

To evaluate the interprofessional course four questionnaires were completed before and after the course: (1) course content Knowledge, (2) Confidence, (3) Attitudes, and (4) Clinical Practice. The questionnaires were modified from those used in previously published studies on oral health interventions for primary care providers (28), and the knowledge questionnaire was created by our study team and faculty lecturers.

The course content Knowledge questionnaire included 24 multiple-choice questions. There were two to three questions designed to cover key points from each of the 10 lectures. The knowledge questionnaire was scored as correct (1) or incorrect (0).

The Confidence questionnaire included 10 items to assess the student’s level of comfort in providing children’s oral health. Each item was rated on a three-level Likert scale, very confident, somewhat confident, or not confident. Responses were coded as 0 for not confident, 1 for somewhat confident, and 2 for very confident. The Cronbach’s alpha coefficient, a reliability coefficient that measures the item’s internal consistency, was 0.95 for the baseline questionnaire.

The Attitude questionnaire included four questions evaluating the student’s attitude toward providing children’s oral health. Each item was rated on a four-level Likert scale, strongly disagree, disagree, agree, and strongly agree. The responses were scored as 0 for strongly disagree, 1 for disagree, 2 for agree, and 3 for strongly agree. The Cronbach’s alpha was 0.80 for the baseline questionnaire.

The Clinical Practice questionnaire included 10 questions evaluating clinical experience and competence of the students’ in providing oral health care to children. Questions 1–3 asked students about how many oral health exams were incorporated into their physical exams, and how many fluoride treatments they performed in the past 3 months. Questions 4–10 asked about “willingness” to perform the items listed. Each item was rated on a four-level Likert scale, frequently, occasionally, rarely, and often. The Cronbach’s alpha was 0.93 for the Clinical Practice questionnaire’s items 4–10.

The data analysis plan was to calculate frequencies and proportions for categorical variables or mean and SD for continuous variables. The demographic data were summarized for each characteristic. The knowledge, confidence, behaviors, and attitude questionnaires were summarized at the item level and as mean scores and compared pre- and post-course. A Wilcoxon ranked test was calculated to compare responses for the pre-test and post-test for mean scores not normally distributed. In order to investigate any change in responses to the individual questions from the pre- to the post-course, the exact McNemar’s test was calculated for dichotomous or categorical responses. Non-parametric and crosstabs were calculated with chi-square analyses.

## Results

A total of 41 students were enrolled in the IPE oral health course over the three quarters. Some of the students did not complete the baseline questionnaire (*n* = 6) or dropped out of the course (*n* = 4) and were excluded from the analysis. In the end, there were a total of 31 students who completed the pre- and post-intervention questionnaires as well as all of the requirements of the intervention. There were 25 students (80%) from the UCSF School of Dentistry (80%), 3 students (10%) from the UCSF School of Nursing, and 3 students (10%) from the Touro University College of Osteopathic Medicine.

The majority of students (Table 2) were between 20 and 21 years of age (78%), female (73%), Asian (61%), and first-generation college students (51%). Twenty seven percent of the students were from underrepresented minority groups and 29% were from disadvantaged backgrounds.

**Table 2:** Students’ demographic characteristics.

Characteristic	Category or response	*N* (*N* = 41)	%
Age (in years)	20–29 30+	32 9	78 22
Sex	Male Female	10 31	24 76
Race	White Hispanic or Latino Asian Black or African-American More than one race	6 5 25 3 2	15 12 61 7 5
Ethnicity	Hispanic or Latino Not Hispanic or Latino	8 33	20 80
Family yearly income	Less than $10,000–$49,000 $50,000–$99,999 $100,000 to more than $150,000	14 14 13	34 34 32
First-generation college student	Yes	21	51
Scholarship	Yes	29	71
Financial aid	Yes	25	61
Loan	Yes	29	71
Underrepresented minority	Yes	11	27
Disadvantaged background	Yes	12	29
Rural residential background	Yes	8	20
Primary language	English Spanish Mandarin Other	32 1 4 4	78 2 10 10
Location of birth	California, USA Another state in USA Another country than USA	17 5 19	42 12 46

### Oral Health Knowledge

Sixty-three percent of the students stated they had formal training in oral health prior to IPE oral health course. The students’ oral health knowledge significantly improved from the pre-to the post-tests [mean (SD) = 15.10 (2.09) and 16.58 (2.90), respectively pre- and post-tests; Wilcoxon signed ranks test, *p* = 0.005], with a moderate effect size (Cohen’s *d* = 0.59) (Table 3).

**Table 3:** Level of students’ knowledge pre- and post-course.

	Knowledge questions	Total number	Pre-test		Post-test		*p*
			Number correct	%	Number correct	%	
1.	In normal dentition, how many primary baby teeth do children have?	31	30	97	28	90	0.500
2.	What is the #1 chronic childhood disease?	31	28	90	31	100	0.250
3.	Which is the right sequence of eruption in primary teeth?	26	18	69	15	58	0.453
4.	What is poor oral health associated with?	31	27	87	28	90	1.000
5.	According to the academy of General Dentistry, what percentage of all systemic disease produces oral signs and symptoms?	30	7	23	10	33	0.508
6.	What is the most common presentation of incipient caries without cavitation?	26	22	85	24	92	0.625
7.	Which permanent teeth erupt first?	31	24	77	21	68	0.453
8.	Which teeth in permanent dentition are most common site for caries?	26	26	100	25	96	1.000
9.	What is one of the top 10 greatest public health achievements?	31	27	87	28	90	1.000
10.	First thing to assess in oral exam?	30	16	53	9	30	0.092
11.	What can be used as a sugar substitute in dental cavity prevention?	31	30	97	31	100	1.000
12.	Which snack is least harmful to teeth?	31	23	74	21	68	0.500
13.	By what age should a child first see a dentist?	31	27	87	30	97	0.375
14.	By what age can parent use fluoride toothpaste?	31	18	58	26	84	0.008*
15.	How long should one exclusively breastfeed?	31	19	61	14	45	0.180
16.	What is vertical transmission of bacteria?	31	29	94	29	94	1.000
17.	What is the most common indication to perform a frenectomy in infants?	26	7	27	19	73	<0.001*
18.	What is the major oral health issue with special needs patients?	31	6	19	4	13	0.688
19.	Why may oral hygiene in special needs children be inadequate?	31	30	97	29	94	1.000
20.	How many times a year should one visit the dentist?	31	25	81	29	94	0.219
21.	According to healthy people 2020 how many people are low health literacy?	31	17	55	20	65	0.549
22.	According to Medicaid, low literacy is what reading level?	31	12	39	14	45	0.774
23.	What is oral health literacy dependent on?	31	19	61	23	74	0.344
24.	According to Academy of Pediatric Dentistry, what percent of children under 6 see a dentist?	30	4	13	6	20	0.754

	**Total score**	**Total number**	**Mean (SD)**	**Median**	**Mean (SD)**	**Median**	***p***

		31	15.10 (2.09)	16.00	16.58 (2.90)	17.00	0.005*

*p<0.05.

The two items that had statistically significant increases in knowledge from the pre- and post-tests were: (1) when to use fluoride (from 58 to 84%) and (2) when to perform infant frenectomy (from 27 to 73%) (McNemar test *p* < 0.008, *p* < 0.001, respectively). However, at the post-test less than 50% of the students correctly responded regarding; what is the first thing to assess in oral assessment (30%), what is main oral health problem in special needs patients (13%), and how many children six and under see a dentist (20%).

### Confidence

There was a statistically significant increase in the students’ confidence in their ability to provide oral health services from the pre- versus post-course completion with a strong effect size [mean (SD) = 13.13 (5.89) and 17.09 (4.02), respectively, *p* < 0.001; Cohen’s *d* = 0.79] (Table 4). There was a statistically significant positive improvement in confidence for these content areas: consulting on fluoride supplements (*p* = 0.004), consulting during dental visit during infancy/childhood (*p* = 0.019), examining teeth of infants and toddlers for tooth decay (*p* = 0.028) and identifying tooth decay in early childhood (*p* = 0.001). Unfortunately, two students were not confident in knowing when to refer a child to the dentist. There was an overall decrease in the percentage of responding “not confident” on the pre-test compared to the post-test.

**Table 4:** Level of students’ confidence pre- and post-course.

		Pre-test						Post-test						
	Confidence	Very confident (2)	%	Somewhat confident (1)	%	Not confident (0)	%	Very confident (2)	%	Somewhat confident (1)	%	Not confident (0)	%	*p*
1.	Consult on child’s oral hygiene	15	47	16	50	1	3	26	81	6	19	0	0	0.191
2.	Consult on water fluoridation	16	50	13	41	3	9	28	88	4	13	0	0	0.090
3.	Dietary consult to prevent early childhood tooth decay	18	56	11	34	3	9	28	88	3	9	1	3	0.365
4.	Consult on fluoride supplement during infancy/childhood	12	39	15	48	4	13	25	81	6	19	0	0	0.004*
5.	Consult on dental visits during infancy/childhood	21	66	9	28	2	6	26	81	6	19	0	0	0.019*
6.	Examining teeth of infants and toddlers for tooth decay	17	53	9	28	6	19	20	63	10	31	2	6	0.028*
7.	Identifying tooth decay in early childhood	13	41	12	38	7	22	22	69	8	25	2	6	0.001*
8.	Identifying other signs of oral pathology	9	28	12	38	11	34	20	63	7	22	5	16	0.105
9.	Evaluating the risk of tooth decay in infants and toddlers	15	47	8	25	9	28	25	78	6	19	1	3	0.064
10.	Deciding if the child needs referral to a dentist	15	48	10	32	6	19	22	71	7	23	2	6	0.075

	Total score	Mean (SD) = 13.13 (5.89); Median = 14.00						Mean (SD) = 17.09 (4.02); Median = 19.00						<0.001*

*p<0.05.

### Attitude and Clinical Practice

Overall there was an increase in the students’ positive attitudes about oral health knowledge from the pre- versus post-course, but it was not statistically significant and had a weak effect size [mean (SD) = 11.10 (1.45) and 11.32 (1.30), respectively, Cohen’s *d* = 0.16] (Table 5). There was a statistically significant difference in the response to the item: prescription of fluoride supplements when indicated (*p* = 0.028). The scores increased positively toward providing oral health care to children. Most of the students wanted to provide preventive oral health care, but one student showed a lack of confidence in providing routine dental check-up at well child visits when starting practice.

**Table 5:** Level of students’ attitudes and clinical practice pre- and post-course.

		Pre-test				Post-test				
	Attitude	Strongly agree (3)	Agree (2)	Disagree (1)	Strongly disagree (0)	Strongly agree (3)	Agree (2)	Disagree (1)	Strongly disagree (0)	*p*
1.	Routine assessment for early signs of dental problems (e.g., dental decay, gingivitis) during the physical exam	24	7	0	0	27	3	1	0	0.144
2.	Referral to dentist by 1 year of age	22	9	0	0	26	5	0	0	0.096
3.	Counseling on the prevention of dental problems (e.g., dental decay, gingivitis, trauma)	26	5	0	0	28	3	0	0	0.394
4.	Prescription of fluoride supplements when indicated	25	5	1	0	23	8	0	0	0.028*

	Total score	Mean (SD) = 11.10 (1.45); Median = 12.00				Mean (SD) = 11.32 (1.30); Median = 12.00				0.393

	**Clinical practice**	**0**	**1–10**	**11–20**	**21+**	**0**	**1–10**	**11–20**	**21+**	***p***

1.	Approximately how many routine physical exams have you performed over the past 3 months?	23	3	2	4	20	7	3	2	<0.001*

		**0**	**1–5**	**6–10**	**11–15**	**0**	**1–5**	**6–10**	**11–15**	***p***

2.	Approximately how many oral health exams have you included in your routine examinations in the past 3 months?	11	10	5	6	6	10	4	12	0.204

		**Yes**		**No**		**Yes**		**No**		***p***

3.	Have you applied fluoride varnish as part of your routine examination to children in past 3 months?	14		16		26		4		<0.001*

		**Frequently (3)**	**Occasionally (2)**	**Rarely (1)**	**Never (0)**	**Frequently (3)**	**Occasionally (2)**	**Rarely (1)**	**Never (0)**	***p***

4.	Assess a child’s fluoride intake to determine the need for supplementation	6	4	3	17	11	6	6	7	0.198
5.	Prescribe a dietary fluoride supplement	6	2	3	17	6	3	4	15	0.266
6.	Discuss the use of fluoride toothpaste with parents	8	9	2	11	12	9	4	5	0.406
7.	Inquire whether a child is taking the bottle to bed	5	5	8	12	9	8	3	10	0.132
8.	Counsel parents on the importance of going to a dentist on regular basis	11	4	4	11	15	7	4	4	0.016*
9.	Inquire about mother’s dental health	6	2	5	17	9	6	7	8	0.105
10.	Referred a high-risk patient to a dentist	8	1	7	12	10	7	4	7	0.011*

	Total scores (for 4–10 items above)	Mean (SD) = 7.87 (7.39); Median = 8.00				Mean (SD) = 11.40 (6.85); Median = 11.00				0.005*

*p<0.05.

There was an overall statistically significant with a moderate effect size increase in students’ clinical practice skills from the pre- versus post-course [mean (SD) = 7.87 (7.39) and 11.40 (6.85), respectively, *p* = 0.005, Cohen’s *d* = 0.50] (Table 5). The students became more aware of providing oral health care to children after the IPE oral health course. There was a significant increase in the number of oral health assessments provided during physical exams in the past 3 months (*p* < 0.001), and applying fluoride varnish applications during routine exams (*p* < 0.001). There was also a statistically significant increase in counseling parents on the importance of regular dentist visits (*p* = 0.016) and referring high-risk patients to a dentist (*p* = 0.011). There were 15 students who lacked confidence in prescribing fluoride and 10 students who lacked confidence in asking about the child taking a bottle to bed.

## Discussion

This study’s objective was to evaluate changes in student’s knowledge, confidence, attitude, and clinical practice in children’s oral health after completing an interprofessional practice and education course on children’s oral health. The majority of students (63%) had some type of oral health education before the course; yet, there was an overall increase in knowledge after the course. Most of the students did not have the baseline knowledge on when to use fluoride (58%) and when to perform a frenectomy (27%) on an infant. The student’s pre-course knowledge showed that less than half the students knew what to assess first in an oral examination (oral tissues) (30%), had a lack of knowledge about providing oral health care for special needs patients (13%), and knew about the limited access to dental care for children living in low-income families (20%).

The confidence for the IPE course was noteworthy because the majority of students were confident in the pre-test before the course, but they became even more confident after completing the oral health course. The curriculum in the course created a significant change (*p* < 0.001) in the overall confidence of the students. There was also an almost 50% increase in students in identifying oral pathology after the curriculum was completed from 13 students to 22 students, although the results were not statistically significant. The overall “not confident” response for all questions decreased, which showed a positive trend to an overall increase in their confidence to perform more oral health procedures and deciding what treatment to provide.

Students’ attitudes about how they felt about pediatric oral health and its overall importance increased although it was not statistically significant. Since the majority of students had some oral health knowledge, the majority found oral health to be important and they had a positive attitude about providing oral health care. The students’ clinical practice had an overall significant increase in routine oral assessments over 3 months. The response of “never” decreased after the intervention, but unfortunately it was still a response for some of the clinical practice questions. Some students were not confident in prescribing fluoride in clinical practice (54%). Future studies should incorporate a mechanism to include feedback about the clinical skills practice to identify ways to encourage more positive attitudes in clinical practice. These findings are similar to an earlier study at UCSF on Oral Health Education for Pediatric Nurse Practitioner Students (28). There was an overall increase in knowledge, confidence, attitude, and clinical practice after completing the curriculum on children’s oral health, but this was not an interprofessional course (28).

A recent study aimed to assess the usefulness of an IPE clinical simulation and case study experience for nurse practitioner (NP)/midwifery (MW), medical, and dental students using oral systemic health and they included self-reported completion of interprofessional competencies (31). A total of 318 students participated in the IPE experience in 2013 and 300 students in 2014. The three-day experience included 100 NP/MW, dental, and medical students participating each day. Before the pre-test and IPE experience, the researchers asked the participants to complete two modules (The Relationship of Oral to Systemic Health and The Oral Examination) and to watch a 9-min video about the IPE competencies. During the standardized patient encounter, teams of four students (one NP or MW, one dental, and two medical students) met in a simulation center exam room with one standardized patient. There was a 60-min session that was facilitated by an NP/MW or MD faculty member; a DDS facilitator toggled between two exam rooms. The physical examination of three organ systems: oral, cardiac, and pulmonary was addressed in the session. All students had a faculty-facilitated session that helped them prepare to provide their respective teaching/teach-back element of the experience. The dental student taught the oral exam, the NP/MW student taught the pulmonary exam, and the medical student taught the cardiac exam, with teach-backs by each student. There was a statistically significant change in student’s mean scores from pre-test to post-test. The faculty facilitators completed a post-test encounter questionnaire that assessed their attitudes about IPE and the value of the IPE experience and the trained faculty facilitators across disciplines reported a high level of agreement that IPE positively influenced students’ interprofessional communication, collaboration, patient communication, and understanding of professional roles and responsibilities. The study findings also supports that oral systemic health IPE is a positive intervention for facilitating medical–dental collaboration and interprofessional training, particularly in oral health promotion and disease prevention. This study had similar findings to ours, but with a different approach in methodology. In this study, each student taught the specifics of their respective fields, while our study included collaboration and discussions among the students as a part of each lecture and during clinical experiences.

The most recent study by Berkowitz and colleagues developed an interprofessional curriculum in partnership with a dental school to teach oral health in the primary care setting to Physician Assistant (PA) students in order to measure the impact of a curricular model that would be easy to adapt across academic settings (32). Twenty-three students over three semesters attended didactics in the classroom, participated in a clinical skills lab, observed in the dental clinic, and observed organized clinical examinations, which were used to teach oral health to first-year PA students. Pre- and post-intervention test results concluded that a short, concentrated amount of instructional time in oral health curriculum had a substantial impact on the retention rate of oral health knowledge for the PA trainees and students express enthusiasm to begin using oral health skills. A concentrated interprofessional oral health program can be successfully integrated into academic settings with an optimistic effect on knowledge and improved patient oral health care. This study is very similar to ours, but it lacks the clinical component that encourages students to provide oral health exams and treatment. This study’s conclusion presented parallel findings. Interprofessional practice and education on oral health will raise awareness in primary care providers and encourage more oral health treatments and timely referrals.

Although this is a novel course, including didactic, discussion, and clinical practice, there were limitations to the study results. There was no control or comparison group who did not participate in an interprofessional oral health course. There were also many correct knowledge responses during the pre-test that could be due to the majority of the students who had some type of oral health education prior to the intervention. The curriculum could be modified to expand the students’ knowledge rather than reinforce knowledge. The small sample size limits our ability to find statistically significant findings yet the course is ongoing so in a few years there will be over 40 more students enrolled in the course. This sample size also limited the ability to compare results in relation to the broader population of health professional students. It is not clear if the changes that occurred as a result of the course would not have occurred without the IPE component. It was not possible to identify differences in knowledge gained by discipline (i.e., dentistry, nursing, medicine).

The interprofessional course included some unique components that helped make it successful. The faculty who taught in the course were from the UCSF Schools of Dentistry, Medicine, and Nursing. The courses in the current nursing and dental curriculum do not consistently include faculty from different disciplines; therefore, this course provided a unique experience and exposure to interprofessional practice and education not included in their traditional program. In addition, the clinical experience for the nursing students was enriched since they attended a dental clinic with a dentist and learned how to conduct an oral health assessment along with dental students. Likewise, the dental students had never attended a clinical practicum with nursing students. There are several studies that showed health professional students exposed to interprofessional clinical and educational experiences are more likely to hire or work collaboratively with professionals from other disciplines after their training (28, 32).

This project includes a follow-up study that will track the students over 5 years to learn about their clinical practice and evaluate if they are using the knowledge gained from participating in the IPE oral health course. Over time, we may be able to determine if and how the course impacted the student’s clinical behavior after graduation.

## Conclusion

To conclude, this study showed that offering an interprofessional course on children’s oral health to graduate students in dentistry, nursing, and osteopathy can improve their knowledge, confidence, and practice toward children’s oral health and expand their professional goals to include caring for underserved, minority children. The pre-test response results displayed their lack of knowledge and confidence in providing oral health care to children. After the course, the majority of the students increased their oral health knowledge and confidence toward providing oral health care to children. Although overall clinical practice behaviors improved, there were still some students who were not ready to provide oral health care to children. Primary care providers are on the forefront of being able to provide anticipatory guidance to parents. They anticipate changes in children’s oral health needs based on the children’s developmental stages (26). Parental dependency, demographic, and environmental context also play a key role in predicting what a child may need for oral health care, and primary care providers are on the forefront to help with oral health prevention through anticipatory guidance to improve oral health outcomes (26). The primary care provider plays an essential role in addressing oral health disparities for young children in the U.S. (20). This course provides students with a foundation for collaborative practice in the community to increase awareness of their respective fields and decrease oral health disparities. Healthcare providers are encouraged to participate in interprofessional practice and education courses and to collaborate across disciplines to deliver high-quality oral health care.

## Ethics Statement

This study was carried out in accordance with the recommendations of the UCSF Committee on Human Research with written informed consent from all subjects. All subjects gave written informed consent in accordance with the Declaration of Helsinki. The protocol was approved by the UCSF Committee on Human Research.

## Author Contributions

DC: lecturer for the course, wrote the manuscript, and worked on the team to develop the evaluation measures, collected measures, and analyzed the data. JK: worked on the team to develop the evaluation measures, developed the online data collection measures, analyzed the data, and reviewed the manuscript. KD: lecturer in the course, developed course objectives for her lecture, and reviewed and edited the final manuscript. RS: lecturer in the course, developed course objectives for his lecture, and reviewed the manuscript. BL: principal investigator on the HRSA grant, developed course objectives for his lecture, lecturer in course, and reviewed the manuscript. AA: lead on the evaluation project, designed the evaluation, supervised and led the analysis team, and edited the final manuscript.

## Conflict of Interest Statement

The authors declare that the research was conducted in the absence of any commercial or financial relationships that could be construed as a potential conflict of interest. The reviewer, LY, and the handling editor declared their shared affiliation, and the handling editor states that the process nevertheless met the standards of a fair and objective review.
